# Histology of an undisplaced femoral fatigue fracture in association with bisphosphonate treatment: Frozen bone with remodelling at the crack

**DOI:** 10.3109/17453674.2010.492766

**Published:** 2010-07-16

**Authors:** Per Aspenberg, Jörg Schilcher, Anna Fahlgren

**Affiliations:** Department of Orthopaedics, IKE, Faculty of Health Sciences, Linköping UniversitySweden

## Introduction

A 57-year-old woman stumbled over her carpet, almost fell, and tried to sit down on the floor, when she heard a crack and sustained a transverse diaphyseal fracture of her left femur. The fracture had been preceded by thigh pain for a couple of months, but this was thought to be caused by spinal stenosis, for which she had been operated twice. She had been diagnosed with seronegative rheumatoid arthritis 10 years before the fracture and had initially undergone different pharmacologcal treatments, all of which except cortisone had been terminated. The diagnosis was repeatedly doubted, but cortisone gave symptomatic relief and was therefore continued at 5 mg/day with intermittent high-dose treatment (25 mg/day) during exacerbations. Apart from prednisolone, no anti-rheumatic drugs had been given during the previous 6 years. The patient had been given alendronate (70 mg/week) in 2001, followed by risedronate (35 mg/week) from 2002 until the fracture in 2009. The patient had also been taking 20–40 mg omeprazol a day since 2000.

**Figure 1.**
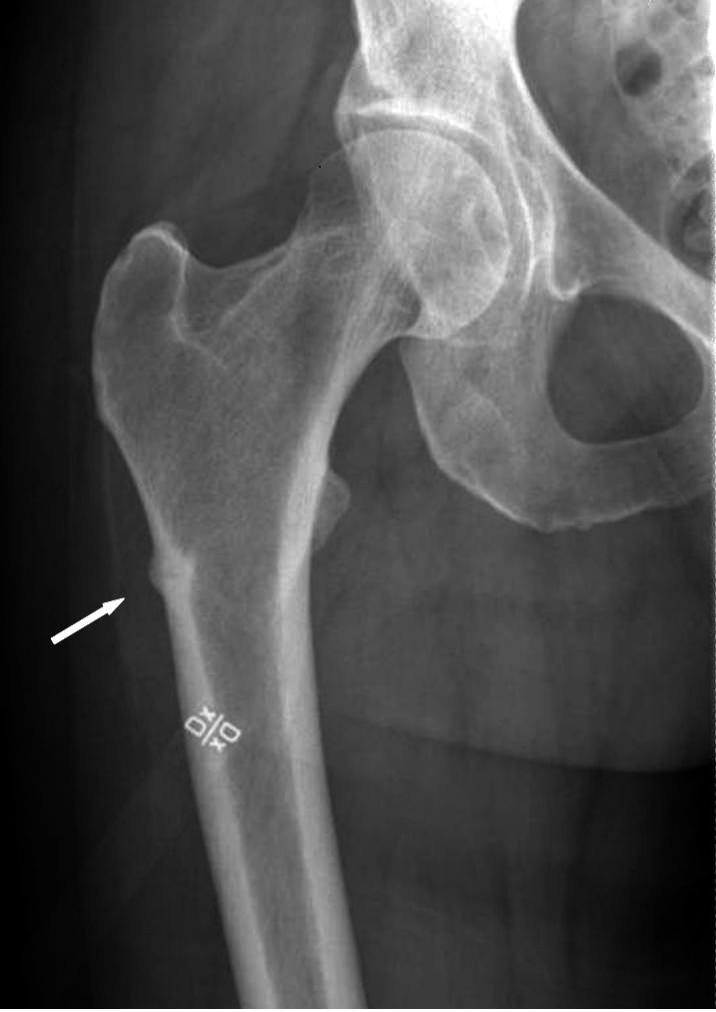
Right femur. Arrow indicates undisplaced fatigue fracture.

The fracture had a typical fatigue fracture appearance (Neviaser et al. 2008), and was operated on with intramedullary nailing. Because of a history of pain on weight bearing also in her contralateral thigh, new radiographs were taken and showed a non-displaced stress fracture of the subtrochanteric region (Figure 1). This fracture was also treated with intramedullary nailing.

### Surgery (second operation)

After the nail had been inserted, the non-displaced fracture was exposed. It could be seen as a dark line the size of a hair on the bone surface, surrounded by a barely visible protrusion of the bone. A 12 × 15-mm specimen including the fracture was excised, with the patient's informed consent. The procedure was approved by the Regional Ethics Committee. Both fractures healed uneventfully and mineralized callus was seen at the biopsy site after 5 months.

### Histology

The specimen was divided in halves. The frontal half was prepared for visualization of microcracks (Burr and Hooser 1995) but this failed for technical reasons. The dorsal half of the specimen was demineralized, embedded in paraffin, sectioned in the sagittal plane, parallel to the periosteal surface, and stained either with HE or with an anti-TRAP antibody (a gift from G Andersson, Karolinska Institute, Stockholm) to identify osteoclasts.

Sections parallel to the cortical surface were analyzed at every 0.5 mm from the periosteum to the endosteal callus. The intact bone at 7 mm distance from the fracture showed a regular osteonal structure with osteocyte lacunae, but very few of these lacunae contained osteocytes. The bone matrix showed paler staining than the bone rich in live osteocytes, seen at other sites (see below). Some osteocyte lacunae in the osteocyte-less bone were indistinctly delineated. There were numerous irregular small cracks in the matrix. These cracks were only seen in osteocyte-less matrix, and not in the younger osteocyte-rich bone. Occasionally, a wide vascular canal with some bone formation was seen within the regions without osteocytes.

**Figure 2.**
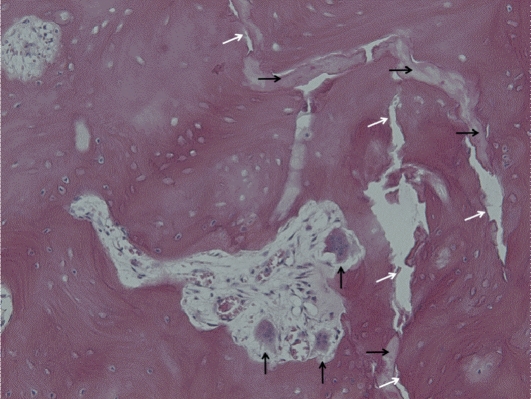
Histology of the fracture, seen both as a vertical crack through the image (white arrows) and as areas of diffusely damaged and disintegrated bone (black horizontal arrows). A resorption cavity with giant detached osteoclasts (black vertical arrows) and new-formed bone with rounded osteocytes can be seen near the fracture.

The fracture appeared mostly as a meandering empty crack, only 0.1 mm wide or less. In some areas, it appeared as a band of necrotic material (Figure 2), consisting of diffusely damaged and disintegrated bone, with a sharp border to the surrounding bone. Fibrous or cartilaginous tissue was never seen within the crack or adjacent to it, and no vessels were seen to approach it. In contrast to the bone at a greater distance from the crack, the bone in its vicinity, inside the femoral cortex, contained numerous resorption cavities with loose marrow. Many of these contained large osteoclasts (Figure 2), sometimes with extremely large numbers of nuclei and with detachment from the underlying bone surface in accordance with findings described by Weinstein et al. (2009). Here the bone was rich in osteocytes, which were often rounded in shape. There were few empty lacunae. The bone had lost its regular osteonal architecture, and remodeling appeared to have occurred in all planes and directions. Remnants of mature osteonal bone were seen interspersed with this irregular young bone. In the old bone, almost all osteocyte lacunae were empty.

There were only minor differences between sections at different depths from the outer surface within the cortex. However, adjacent to the bone marrow there was an endosteal callus with very little necrotic bone, but instead a dense remodeled bone with an irregular osteonal structure and vivid ongoing remodeling. The fracture crack terminated within this new bone. Near the periosteal surface there were no differences from the interior of the cortex, except superficially, where a thin layer of immature bone (callus) was seen.

Osteocytes were counted in 3 sections, 0.5 mm apart, corresponding to approximate depths of 1.5, 2, and 2.5 mm from the periosteal bone surface. The measured area in each section was a 1 × 5-mm rectangle, oriented parallel to the fracture, about 7 mm away from it. Of 1,967 lacunae counted, only 488 of them (25 %%) contained visible cellular material. As a control, new-formed bone in the endosteal callus was analyzed in a similar way, which showed that 86% of the lacunae contained cellular material, despite the fact that some necrotic bone was included in the area analyzed.

Not a single multinucleated TRAP-positive cell was identified in the measuring area 7 mm away from the crack, whereas there were 7 such cells per mm^2^ of specimen surface in the fracture region (385 cells counted).

## Discussion

Stress fractures in association with bisphosphonate treatment have attracted attention recently (Aspenberg 2009). It appears that in this case the fracture had occurred in bone that was almost completely devoid of osteocytes. However, the cortical bone was able to undergo osteoclastic resorption adjacent to the fracture, showing that remodeling was possible. Targeted remodeling is thought to be initiated by signals from dying osteocytes, and it thus requires these cells to be present. Either the remaining small numbers of osteocytes were sufficient or some other mechanism may have initiated resorption.

The abundant, diffusely damaged and disintegrated bone in the fracture region is puzzling (Figure 2). It does not fit with the common description of diffuse microdamage (Burr, personal communication). Although it might have caused the fracture, its location—almost exclusively at the crack—suggests that it could have been secondary to the fracture.

Somford et al. (2009) recently described markedly increased resorption within a cortical biopsy taken about 1 cm from the fracture in a similar case. Their findings suggest that the bone was weakened due to resorption. In our patient, however, resorption was limited to the immediate vicinity of the fracture and was obviously a consequence of it, and the fracture ran through areas of necrotic bone.

Long-term bisphosphonate treatment dramatically increases the risk of femoral stress fracture, although the annual risk is still only about 1 in 1,000 (95% CI: 0.2–2) (Schilcher and Aspenberg 2009). It appears that both cortisone and proton pump inhibitors can increase the risk (Kwek et al. 2008, Ing-Lorenzini et al. 2009), and our patient was taking both of these drugs as well. The lack of living osteocytes in the central femoral cortex suggests that the bone had become too old for osteocyte survival, due to severe suppression of remodeling, or that corticosteroids had induced osteocyte apoptosis, or probably both. Stress fractures are thought to be initiated by microcrack accumulation in bone that has not undergone timely remodeling, or which has become highly mineralized and brittle for the same reason.

Stress fractures heal slowly. Still, in our case, the cortical bone near the crack was able to undergo intense remodeling in spite of its bisphosphonate content. Even though some osteoclasts may have shown pathological histological features such as detachment and giant size, their number and activity was sufficient for extensive remodeling. This suggests that non-healing of the fracture and its possible progression to gross failure was related to the mechanics or geometry of the crack, and not to qualities of the surrounding bone or the patient's ability to heal. It was striking that the crack did not contain any new-formed tissue. A speculative explanation would be that because the crack was so narrow, even a small physiological strain that would widen or compress the crack by fractions of a millimeter would cause a local strain sufficient to tear apart any cell or vessel daring to enter the area. Thus, although our patient's fracture may have been initiated in “frozen bone” due to bisphosphonate treatment, its inability to heal may have had similar causes to those for stress fractures in general.

Although this patient showed a striking lack of osteocytes, our findings do not explain the detailed mechanisms behind initiation of the fracture. In order to do that, one would have to study biopsies from this region, before the fracture has occurred, with specific methods to visualize microcracks and diffuse microdamage.
